# Genome Sequence of *Oceanicola* sp. Strain MCTG156(1a), Isolated from a Scottish Coastal Phytoplankton Net Sample

**DOI:** 10.1128/genomeA.00796-17

**Published:** 2017-08-10

**Authors:** Tony Gutierrez, William B. Whitman, Marcel Huntemann, Alex Copeland, Amy Chen, Neha Vargese, Nikos C. Kyrpides, Manoj Pillay, Natalia Ivanova, Natalia Mikhailova, Supratim Mukherjee, Dimitrios Stamatis, T. B. K. Reddy, Chew Yee Ngan, Mansi Chovatia, Chris Daum, Nicole Shapiro, Tanja Woyke

**Affiliations:** a School of Engineering and Physical Sciences, Heriot-Watt University, Edinburgh, United Kingdom; b Department of Microbiology, University of Georgia, Athens, Georgia, USA; c DOE Joint Genome Institute, Walnut Creek, California, USA

## Abstract

*Oceanicola* sp. strain MCTG156(1a) was isolated from a phytoplankton net sample collected on the west coast of Scotland and selected based on its ability to degrade polycyclic aromatic hydrocarbons. Here, we present the genome sequence of this strain, which comprises 3,881,122 bp with 3,949 genes and an average G+C content of 62.7%.

## GENOME ANNOUNCEMENT

*Oceanicola* sp. strain MCTG156(1a) was isolated from a phytoplankton net sample that was trawled in 2009 at a sampling station designated LY1, located on the west coast of Scotland near Oban, Argyll. The strain was isolated by enrichment with phenanthrene in Zobell’s 2216 marine medium at 10-fold dilution. Colonies on agar plates sprayed with phenanthrene produced distinct halos that indicated the strain’s ability to degrade the hydrocarbon. Based on 16S rRNA gene sequence identity, the closest type species was *Oceanicola pacificus* strain W11-2B^T^, which had been isolated from a pyrene-degrading consortium that was enriched from sediment from the Pacific Ocean (1).

Here, we report the genome sequence of *Oceanicola* sp. strain MCTG156(1a). Genomic DNA was sequenced through the DOE Joint Genome Institute (JGI) 2014 Genomic Encyclopedia of Type Strains, Phase III study (2), using the Pacific Biosciences (PacBio) technology. A PacBio SMRTbell library was constructed and sequenced on the PacBio RS platform, which generated 239,103 filtered subreads totaling 750.9 Mb. All general aspects of library construction and sequencing performed at the JGI can be found at http://www.jgi.doe.gov. The raw reads were assembled using HGAP version 2.1.1 (3). The final draft assembly produced 5 scaffolds containing 5 contigs totaling 3.9 Mb in size with an input read coverage of 217.9×.

Project information is available in the Genomes OnLine Database (4). Genes were identified using Prodigal (5), as part of the JGI’s microbial annotation pipeline (6). The predicted coding sequences (CDSs) were translated and used to search the NCBI nonredundant, UniProt, TIGRFam, Pfam, KEGG, COG, and InterPro databases. The tRNAScanSE tool (7) was used to find tRNA genes, whereas rRNA genes were found by searches against models of the rRNA genes built from SILVA (8). Other noncoding RNAs, such as the RNA components of the protein secretion complex and RNase P, were identified by searching the genome for the corresponding Rfam profiles using Infernal (http://infernal.janelia.org). Additional analysis and manual functional annotation was performed within the Integrated Microbial Genomes—Expert Review (IMG ER) platform (https://img.jgi.doe.gov) developed by the JGI (Walnut Creek, CA, USA) (9).

The complete genome sequence length was 3,881,122 bp with a G+C content of 62.7%. The genome contained 3,949 genes (3,881 protein-coding genes) with functional predictions for 3,226 of them. A total of 68 RNA genes were detected. Other genes, characteristic for the genus, are given in the IMG database (10).

### Accession number(s).

The draft genome sequence of *Oceanicola* sp. strain MCTG156(1a) obtained in this study was deposited in GenBank as part of BioProject no. PRJNA224116, with individual genome sequences submitted as whole-genome shotgun projects under the accession no. JQMY00000000.
